# Planned vs. rescue rotational atherectomy in severe coronary calcification: procedural complications and one-year clinical outcomes

**DOI:** 10.3389/fcvm.2025.1599091

**Published:** 2025-06-19

**Authors:** Xiaogang Liu, Lei Wan, Yufeng Liu, Ye Gu, Liqun Hu

**Affiliations:** Department of Cardiology, Wuhan Fourth Hospital, Wuhan, China

**Keywords:** planned rotational atherectomy, rescue rotational atherectomy, outcome, severe calcified lesions, major adverse cardiac and cerebrovascular events (MACCE)

## Abstract

**Objective:**

Current guidelines recommend rotational atherectomy (RA) as a rescue treatment for calcified or fibrotic lesions that cannot be fully expanded before stent implantation. Present study compared the procedural and one-year clinical outcome of planned (pRA) or rescue RA (rRA) for patients undergoing percutaneous coronary intervention with severe coronary stenosis and calcification.

**Methods:**

A total of 111 consecutive patients who underwent RA at the Fourth Hospital of Wuhan from July 2021 to June 2023 were enrolled. The general clinical data, coronary artery lesion characteristics, procedural characteristics, complication rate and major cerebral and cardiovascular event [MACCE, cardiac death, acute myocardial infarction (AMI), target vessel revascularization or acute ischemic stroke] rate at one year after procedure were compared between the two groups.

**Results:**

According to the timing of initiation of RA, patients were stratified into pRA group (*n* = 84) or rRA group (*n* = 27). Baseline clinical characteristics were similar between the two groups. The number of stents implanted was similar in the two groups. The rRA group required more pre—dilation balloons (1.7 ± 0.7 vs. 3.4 ± 0.5, *P* < 0.001), exhibited a higher rate of coronary artery dissection (29.6% vs. 7.1%, *P* = 0.02) and consumed a larger volume of contrast (189.8 ± 59 ml vs. 139.9 ± 46 ml, *P* < 0.001). Additionally, the incidence of contrast—induced nephropathy was significantly greater in the rRA group (29.6% vs. 9.5%, *P* = 0.01), and the procedure duration was markedly longer in this group compared to the pRA group (91.5 ± 24.3 min vs. 77.9 ± 25.2 min, *P* < 0.001). Multivariable logistic regression identified rRA as an independent predictor of periprocedural complications (adjusted OR = 2.83; 95% CI:1.01–7.99; *P* = 0.048). However, 1-year MACCE rates showed no intergroup difference (pRA 3.7% vs. rRA 4.8%; *P* = 1.00). No significant difference in the secondary endpoints of non-cardiac death, angina pectoris, heart failure, and cardiovascular rehospitalization were observed between the two groups.

**Conclusion:**

rRA is related with higher procedural complication rates, procedure time, and contrast agent dose compared with pRA, but has similar low MACCE rate as pRA at one year after procedure.

## Graphical Abstract

**Figure:**
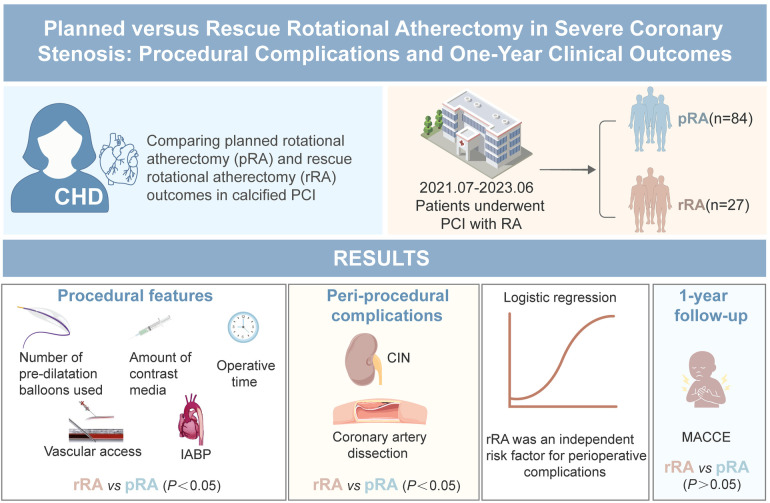

## Introduction

Coronary atherosclerotic heart disease (CAD) has become one of the major diseases that endanger people's health worldwide. With the development of interventional treatment technology, percutaneous coronary intervention (PCI) has become the most widely used treatment method for CAD, but severe coronary artery calcification (CAC) lesions remain as difficult challenge for the success of PCI (1–4). PCI in severe calcified coronary artery stenosis is often linked with serious problems such as low success rate, high incidence of complications, high incidence of postoperative restenosis and cardiac events, and poor prognosis (2, 5). For CAC, rotational atherectomy (RA) can reduce procedural complications and improve the success rate of PCI (6, 7). RA for CAC is usually divided into the following two situations: (1) severe calcification is visualized by coronary angiography or intravascular ultrasound (IVUS), RA is decided as a pre-treatment method for the lesion, which is planned rotational atherectomy (pRA); (2) RA was used as an “emergency rescue” procedure in case of difficulties occurred in balloon or stent delivery, or when the balloon cannot be effectively expanded or the stent fails to pass, which is rescue rotational atherectomy (rRA) (8).

The European Society of Cardiology guidelines for coronary artery revascularization highlight the necessity of using RA in patients with significant stenosis and calcified lesions (9). Due to the limitations of coronary angiography for calcified lesions, especially calcified nodules, it is sometimes difficult to judge whether it is necessary to start RA to pretreat calcified lesions. Sometimes, it is necessary to switch to rRA during the preparation of routine balloon dilatation before attempting coronary stent implantation. Studies have shown that pRA can reduce the incidence of adverse cardiovascular events at 1 year compared with rRA (8). This benefit may be related to the ability to perform effective balloon dilatation, reduce intraoperative complications, and obtain sufficient lumen area after pRA. However, some studies have shown that pRA does not improve the clinical efficacy of patients compared with rRA when the balloon cannot be fully expanded (10, 11). Therefore, it is of clinical significance to compare the impact of pRA and rRA on procedural and clinical outcomes. This study compared the procedural outcome and clinical outcome at 1 year after pRA or rRA in a realworld patient cohort.

## Materials and methods

### Study subjects

This study was a single-center, retrospective study. A total of 111 patients who underwent RA in the Department of Cardiology of our hospital from July 2021 to June 2023 were enrolled. These patients had a preoperative diagnosis of stable angina, unstable angina, or non-ST-segment elevation myocardial infarction. All patients signed the informed consent before PCI. Exclusion criteria: (1) acute ST-segment elevation myocardial infarction; (2) bridging vessel lesions; (3) acute thrombotic lesions; (4) in-stent restenosis lesions.

### Indications for intracoronary Ra

The diagnosis of coronary artery calcification mainly relies on imaging methods, and the commonly used methods are coronary artery CT examination, coronary angiography and IVUS. The main indications include (12): (1) severe calcification, that is, clear coronary artery calcification shadows can be seen before contrast injection; (2) when coronary angiography cannot determine whether the lesion vessel is severely calcified, IVUS is used to determine the degree of coronary artery calcification. Lesions with a range of >270° are severely calcified; (3) Calcified lesions that cannot be fully pre-dilated by the balloon.

### Clinical and angiographic data

(1) Clinical related indicators: sex, age, cardiovascular risk factors were noted, renal function, blood lipids, serum troponin I (TnI) and creatine kinase-isoenzyme (CK-MB) were measured at admission and 24 h after RA. Left ventricular ejection fraction (LVEF) was measured by echocardiography at admission. (2) Coronary angiography related indicators including severity of coronary artery lesions were analyzed. (3) Surgical operation related indicators including target vessel of RA, diameter of RA head, number of balloons used before stent placement, number of stents, total length of stents, operation time, amount of contrast agent used and incidence of intraoperative related complications were analyzed.

### Revascularization methods and processes

PCI was performed through the radial artery, brachial artery or femoral artery, and unfractionated heparin 70–100 U/kg was given before PCI. The Boston Scientific Rotablator TM was used for RA, and the RA head was Rota Link TM (diameters: 1.25 mm, 1.50 mm, and 1.75 mm, respectively). The size of the RA head was selected to have a ratio of 0.5–0.6 between the RA head diameter and the vessel diameter, and the RA speed was 140,000–160,000 rpm, and each RA lasted 10–15 s. During RA, continuous intracoronary infusion of a mixture containing unfractionated heparin and nitroglycerin was used. The success of RA was defined as complete balloon dilatation of the target lesion after RA. Before RA, all patients received 300 mg aspirin and an oral loading dose of a P2Y12 inhibitor (clopidogrel or ticagrelor). At discharge, all patients received aspirin (100 mg daily) plus clopidogrel (75 mg daily) or ticagrelor (90 mg twice daily) for at least 1 year.

### Perioperative complications

The occurrence of perioperative complications of RA was recorded, including complications directly related to RA and complications not directly related to RA. Complications directly related to RA included: slow coronary artery blood flow or no reflow, coronary artery spasm, severe coronary artery dissection, coronary artery perforation or cardiac tamponade, RA head incarceration that could not be evacuated, and severe bradycardia. Severe coronary artery dissection mainly refers to type C or above dissection defined by the National Institute of Heart, Lung, and Blood Diseases of the United States (13). Complications not directly related to RA refer to PCI- related complications, such as target lesion side branch occlusion, puncture site hematoma, and contrast-induced nephropathy. Angiographic success was defined as final residual stenosis <30% and TIMI blood flow grade III. Perioperative myocardial infarction was defined as TnI exceeding 5 times the upper reference limit within 48 h and new ECG changes or imaging evidence after PCI (14).

### Clinical follow-up

Patients were clinically followed up at 1, 3, 6, and 12 months, and then every 6 months after PCI. The primary endpoint was major adverse cardiovascular and cerebrovascular events (MACCE) were defined as cardiac death, spontaneous myocardial infarction (MI), ischemic stroke, and repeat target vessel/leision revascularization (TVR/TLR). The secondary endpoints were the composite of non-cardiac death, angina pectoris, heart failure and cardiovascular rehospitalization. All endpoints were defined according to the standardized criteria proposed by the Cardiovascular Trials Initiative (15). Deaths were categorized as cardiac or noncardiac, with deaths of unknown etiology classified as cardiac deaths. Cardiac death was defined as death due to cardiovascular causes, including acute myocardial infarction, sudden cardiac death, heart failure (HF), stroke, complications from cardiovascular procedures, cardiovascular hemorrhage, and other cardiovascular-related etiologies. The clinical definition of Ml denotes the presence of acute myocardial injury detected by abnormal cardiac biomarkers in the setting of evidence of acute myocardial ischaemia. Stroke was defined on the basis of the presence of acute infarction as demonstrated by imaging or based on the persistence of symptoms. TVR was defined as any repeat PCI or coronary artery bypass grafting (CABG) of the target vessel. Target lesion revascularization (TLR) was defined as any repeat revascularization of the target lesion with PCI or CABG for restenosis or other complications, which was defined as the treatment segment from 5 mm proximal to 5 mm distal to the stent.

## Statistical analysis

All data were processed using SPSS V.26.0 (IBM SPSS). Normally distributed quantitative data were expressed as x ± s, and the t test was used for inter-group comparison. Non-normally distributed data were expressed as median (interquartile range), and the rank sum test was used for inter-group comparison. Enumeration data were expressed as percentage (%), and the Chi-square (*χ*^2^) test or Fisher's exact probability test was used for inter-group comparison. *P* < 0.05 was considered statistically significant. The effects of various clinical data, including hypertension, diabetes, heart failure, previous PCI, IABP, performance of rRA and preoperative diagnosis on periprocedural complications were evaluated using the Logistic regression module, and the 95% confidence interval (CI) of the odds ratio (odds ratio, OR) and *P* < 0.05 were considered statistically significant.

## Results

### Comparison of baseline data between the two groups

**Figure 1:**
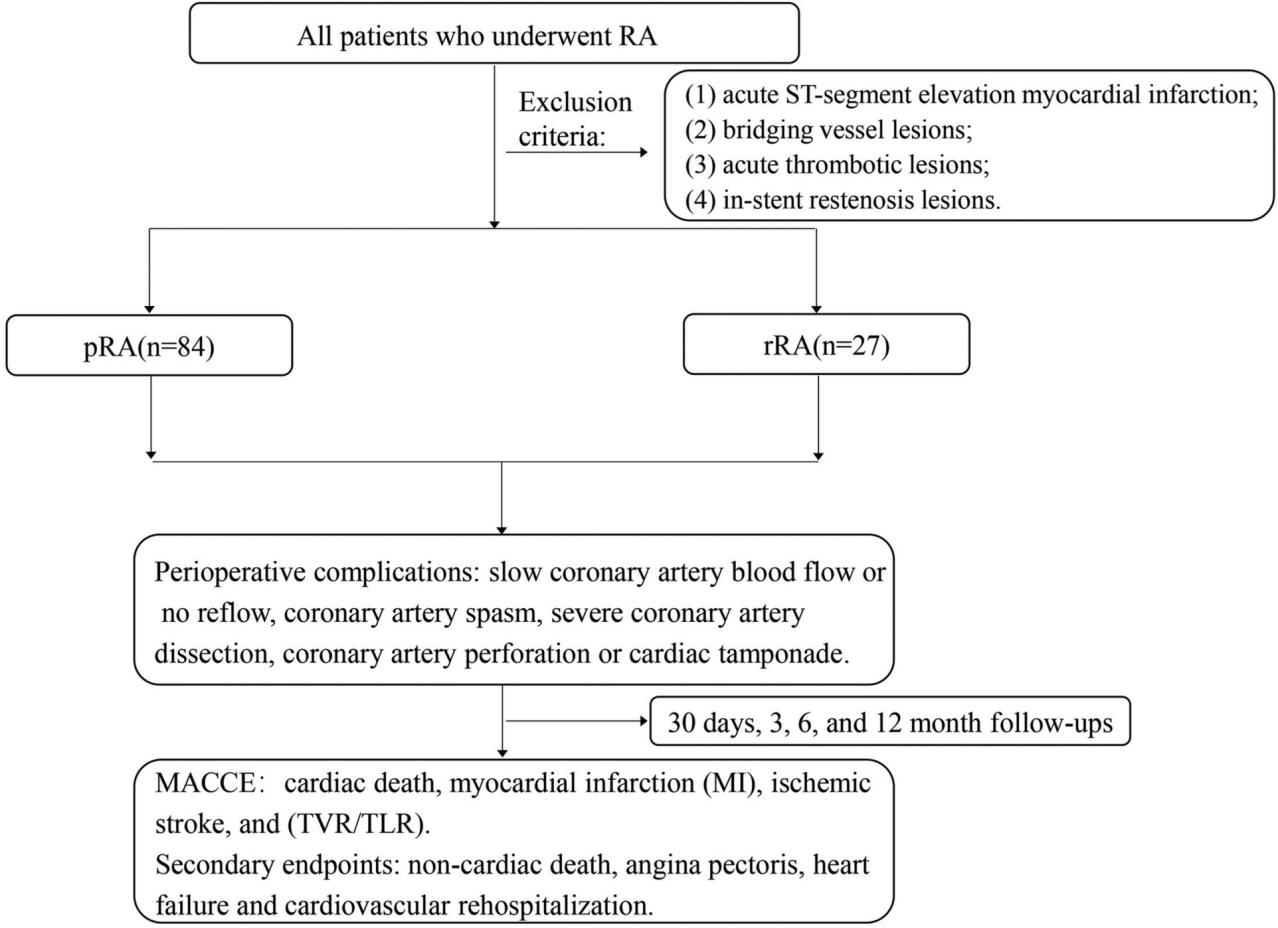
RA, rotational atherectomy; pRA, planned rotational atherectomy; rRA, rescue rotational atherectomy; MACCE, major adverse cardiovascular and cerebrovascular events.

According to the timing of RA, patients were divided into the pRA group (*n* = 84) or the rRA group (*n* = 27) (Figure 1). As shown in Table 1, patients in the pRA group had a higher proportion of dialysis (23.3% vs. 9.5%, *P* = 0.01) and unstable angina (89% vs. 47.6%, *P* = 0.04) (Table 1) than the rRA group, and the rRA group had a higher proportion of non-STEMI (52.4% vs. 11%, *P* = 0.02). There were no significant differences in other baseline clinical characteristics such as sex, age, body mass index, cardiovascular risk factors, cardiac function, and previous medical history (myocardial infarction, PCI) (all *P* > 0.05).

**Table 1:** Baseline characteristics of patients undergoing RA.

Variable	pRA group (*n* = 84)	rRA group (*n* = 27)	*P*
Age, years old	69 ± 9	69 ± 12	0.92
Male, *n* (%)	51 (60.7)	12 (44.4)	0.14
Previous myocardial infarction, *n* (%)	3 (3.6)	2 (7.4)	0.76
Previous PCI, *n* (%)	13 (15.5)	6 (22.2)	0.42
Hypertension, *n* (%)	75 (89.3)	23 (85.2)	0.82
Diabetes, *n* (%)	39 (46.4)	13 (48.1)	0.88
Hyperlipidemia, *n* (%)	20 (23.8)	5 (18.5)	0.57
EF (mean ± s, %)	58.4 ± 6.3	56.2 ± 7.6	0.13
NT-proBNP (mean ± s, pg/ml)	1,868.7 ± 714.2	4,026.3 ± 1,385.7	0.15
NYHA, *n* (%)			
I	53 (63.1)	20 (53.9)	0.30
II	20 (23.8)	5 (18.5)	0.57
III	8 (9.5)	4 (14.8)	0.44
IV	3 (3.6)	4 (14.8)	0.10
HF, *n* (%)			
HFpEF	24 (28.6)	8 (29.6)	0.92
HFmrEF	5 (6.0)	3 (11.1)	0.64
HFrEF	2 (2.4)	2 (7.4)	0.53
Smoking, *n* (%)	23 (27.4)	4 (14.8)	0.28
Renal insufficiency (GFR < 80)	40 (47.6)	12 (44.4)	0.77
Unstable angina, *n* (%)	71 (84.5)	17 (63)	0.04
Non-STEMI, *n* (%)	10 (11.9)	10(37)	0.02

RA, rotational atherectomy; pRA, planned rotational atherectomy; rRA, rescue rotational atherectomy; PCI, percutaneous coronary intervention; EF, ejection fraction; Non-STEMI, non-ST-segment elevation myocardial infarction; NYHA, New York Heart Association; HFpEF, Heart failure with preserved ejection fraction; HFmrEF, heart failure with mildly reduced ejection fraction; HFrEF, heart failure with reduced ejection fraction.

### Comparison of lesion characteristics and procedural features between the two groups

As shown in Table 2, there were no significant differences in target vessels, lesion classification, brachial artery access diameter, femoral artery access diameter, RA head diameter, number of stents implanted, and intraoperative IVUS use between the two groups (*P* > 0.05). The average diameter of the RA head was also similar between the two groups (1.47 ± 0.08 mm vs. 1.38 ± 0.16 mm, *P* = 0.91). The chronic occlusion rate in the rRA group was higher than that in the pRA group (7.1% vs. 22.2%, *P* = 0.03). In terms of PCI approach, radial artery route was more often used in the pRA group (95.2% vs. 81.5%, *P* = 0.02), while femoral artery route was more often used in the rRA group (1.2% vs. 11.1%, *P* = 0.02). The use of IABP during PCI was higher in the rRA group than in the pRA group (19% vs. 51.9%, *P* < 0.001). The number of pre-dilatation balloons used in the pRA group was significantly lower than that in the rRA group [(1.7 ± 0.7) vs. (3.4 ± 0.5), *P* < 0.001]. In the rRA group, the operation time was longer [(91.5 ± 24.3) min vs. (77.9 ± 25.2) min, *P* < 0.001], and the amount of contrast medium used was larger [(189.8 ± 59) ml vs. (139.9 ± 46) ml, *P* < 0.001].

**Table 2:** Analysis of the characteristics of coronary artery lesions and interventional treatment in two groups of patients.

Variable	pRA group (*n* = 84)	rRA group (*n* = 27)	*P*
Target blood vessels			
LM	8 (9.5)	5 (18.5)	0.21
LAD	67 (82.7)	17 (68)	0.11
LCX	4 (4.8)	2 (7.4)	0.60
RCA	10 (11.9)	6 (22.2)	0.18
Chronic occlusive disease	6 (7.1)	6 (22.2)	0.03
Lesion classification			0.88
Single Vessel Disease	8 (9.5)	1 (3.7)	0.58
Multivessel disease	68 (81)	21 (77.8)	0.72
Surgical approach			
Radial artery	80 (95.2)	22 (81.5)	0.02
Brachial artery	3 (3.6)	2 (7.4)	0.40
Femoral artery	1 (1.2)	3 (11.1)	0.02
IABP, *n* (%)	16 (19)	14 (51.9)	<0.001
Intraoperative use of IVUS	28 (33.4)	10 (37)	0.82
Operation time	77.9 ± 25.2	91.5 ± 24.3	0.01
Contrast agent dosage	139.9 ± 46	189.8 ± 59	<0.001
Grinding head diameter	1.47 ± 0.08	1.38 ± 0.16	0.91
Number of pre-dilation balloons used	1.7 ± 0.8	3.4 ± 1.5	<0.001
Number of brackets	1.7 ± 0.8	1.7 ± 0.7	0.51

pRA, planned rotational atherectomy; rRA, rescue rotational atherectomy; LM, left main artery; LAD, descending anterior artery; LCX, circumflex coronary artery; RCA, right coronary artery; IABP, intraaortic balloon pump. IVUS, intravascular ultrasound.

### Analysis of influencing factors of peri-procedural complications

Table 3 showed that incidence of coronary artery dissection and contrast-induced nephropathy was significantly higher rRA group than in the pRA group (both *P* < 0.05). There were no significant differences in other peri-procedural complications (coronary spasm, slow blood flow and no reflow, bradycardia, side branch occlusion and periprocedural myocardial infarction) between the two groups. Peri-procedural complications following coronary RA were designated as the dependent variable, and potential predictors of complications between groups, including hypertension, diabetes, heart failure, previous PCI, IABP, performance of rRA and preoperative diagnosis were included as independent variables. Logistic regression analysis revealed that performance of rRA (OR = 2.834; 95% CI: 1.006–7.986) was an independent predictor of periprocedural complications after RA (*P* < 0.05) (Table 3).

**Table 3:** Multifactorial logistic regression model of peri-procedural complications risk.

Influencing factors	β	SE	WaldX^2^	OR(95%CI)	*P*
Diabetes	0.569	0.449	1.607	1.766 (0.733–4.254)	0.205
Heart Failure	0.493	0.523	0.890	1.638 (0.588–4.563)	0.345
Hypertension	1.830	1.104	2.748	6.232 (0.716–54.217)	0.097
Previous PCI	−0.692	0.666	1.081	0.500 (0.136–1.846)	0.299
IABP	−0.078	0.563	0.019	0.925 (0.307–2.788)	0.890
rRA	1.658	0.528	3.877	2.834 (1.006–7.986)	0.049
UA	−0.179	0.632	0.080	0.836 (0.242–2.889) 0.777	

PCI, percutaneous coronary intervention; IABP, intraaortic balloon pump; rRA, rescue rotational atherectomy; UA, unstable angina.

### MACCE during hospitalization and at one year after PCI between the two groups

All patients completed 1-year follow-up, and MACCE occurred in 4 patients in pRA group and 1 patient in the rRA group (*P* = 0.67). The secondary endpoints were similar between the two groups (22% vs. 33%, *P* = 0.27). There were 4 cases of non cardiac death in both in the pRA group and the rRA group (*P* = 0.18), all related to pulmonary infection (Table 4).

**Table 4:** Perioperative complications and 1-year clinical outcomes in both groups.

Parameter	pRA group(*n* = 84)	rRA group(*n* = 27)	*P*
Perioperative complications			
Coronary artery dissection	6 (7.1)	8 (29.6)	0.02
Coronary artery spasm	17 (20.2)	4 (14.8)	0.53
Slow flow and no-reflow	5 (6.0)	2 (7.4)	0.79
Severe bradycardia	11 (13.1)	3 (11.1)	0.79
Side branch occlusion	2 (2.4)	1 (3.7)	0.71
Periprocedural myocardial infarction	7 (8.4)	3 (11.1)	0.97
Contrast-induced nephropathy	8 (9.5)	8 (29.6)	0.01
The incidence of MACCE during hospitalization	0 (0.0)	1 (9.5)	0.67
MACCE 1 year after PCI	4 (4.8)	1 (3.7)	1.00
Secondary endpoints	19 (22.6)	9 (33.3)	0.27
Unstable angina	13 (15.5)	6 (22.2)	0.42
Arrhythmias	2 (2.4)	0 (0.0)	1.00
Heart failure	6 (7.1)	2 (7.4)	0.96
Cardiovascular readmission	16 (19)	8 (29.6)	0.25
Non-cardiac death	4(2.4)	4(7.4)	0.18

RA, rotational atherectomy; pRA, planned rotational atherectomy; rRA, rescue rotational atherectomy; MACCE, major adverse cardiovascular and cerebrovascular events.

## Discussion

Our findings can be summarized as follows: despite increased peri-procedural complications in the rRA group, the long-term MACCE rate is similarly low as the pRA group. Therefore, rRA is a feasible and similar safe procedure as pRA in the long-term for patients undergoing selective PCI. Thus, there is no need to hesitate the application of rRA in indicated patients based on present results. Clearly, options are required to reduce the peri-procedural complications in rRA patients. Our real-world results are in line with previous meta-analysis (16).

rRA is known to be related to the deviation in the initial judgment of the severity of calcification or fibrous lesions based on coronary angiography. Most of the time, rRA is used after conventional pretreatment of the lesions fails. This transformation is mostly passive, as on the one hand increases the operation time, use of contrast agents and interventional instruments (17). On the other hand failure of the lesion by conventional preconditioning may increase the difficulty and risk of subsequent rotational milling treatment, especially the effect of excessive balloon preexpansion force on the localization of the lesion. Higher balloon pre-dilatation pressure is often required before rRA, and higher pre-dilatation pressure may lead to coronary artery entrapment or even vessel rupture, and even though balloon dilatation appears to be adequate, the stent may still have difficulty in reaching the lesion site. In the contrast, pRA can be used to pre-treat heavy calcified plaques and calcified nodules with RA, which changes the compliance of calcified plaques and facilitates the subsequent interventions, and can effectively avoid the complications associated with forcible pushing of balloons and stents or high-pressure dilation of balloons and stents.

Our results are consistent with above observations. In this study, we found that the percentage of patients in the pRA who developed entrapment was not high, and in fact vascular entrapment was conversely more common when repeated balloon dilatation of the lesion was used (especially for calcified lesions). The number of balloons used for predilatation of lesions (*P* < 0.001), procedure time (*P* < 0.05), and contrast dosage (*P* < 0.001) were significantly lower than in patients treated with rRA after predilatation failure. The reason for this may be that direct high-speed RA of the calcified lesion site makes the serious calcified nodules or calcified plaques removed by the RA size, and the modified and polished wall is relatively conducive to the passage of the balloon or stent, and the IVUS examination found that the calcified ring in the lumen was interrupted to varying degrees after RA, so that the balloon could more easily dilate the lesion, and it was not necessary to use more pre-dilatation balloon, which not only reduced the occurrence of complications, such as entrapment, but also made the procedure smoother. This reduces complications such as entrapment, and also results in a smoother procedure with shorter operative time and lower intraoperative contrast dosage.

Despite the known disadvantage during peri-procedural period (10, 18), the long-term outcome of rRA remains as the research focus of clinical research. We compared the MACCE rate between pRA and rRA, which was similarly low in the two groups (4.8% and 3.7%, *P* > 0.05). The rates of secondary endpoints, the composite of non-cardiac death, angina pectoris, heart failure and cardiovascular rehospitalization, are also similar between the two groups. Recent studies suggest that periprocedural myocardial infarction (MI) has prognostic significance in non-ST-segment elevation myocardial infarction (NSTEMI) (19). However, this association was not observed in our study, potentially because NSTEMI patients constituted only 18% of the cohort. Furthermore, the lack of correlation between higher procedural complications and MACCE in the rRA group may be explained by the following mechanisms: (i) All periprocedural complications were promptly managed through timely interventions, thereby mitigating the risk of complication-related adverse outcomes. (ii) Patients undergoing rRA inherently exhibited higher baseline risks due to complex coronary anatomy and procedural complexity. Nevertheless, RA-mediated plaque modification significantly improved stent expansion and wall apposition, consequently reducing risks of in-stent thrombosis or restenosis and counterbalancing the long-term prognostic impact of rRA. (iii) Intensified postprocedural antithrombotic regimens (e.g., dual antiplatelet therapy with adjunctive anticoagulation), implemented in response to intraprocedural thrombotic risks, further attenuated long-term event risks in the rRA group.

The clinical implications of these findings are twofold. First, meticulous preprocedural evaluation of PCI candidates should include advanced imaging to characterize high-risk lesions. For instance:

IVUS quantifies calcification thickness (threshold >0.5 mm indicating potential need for RA) and arc curvature. Optical coherence tomography (OCT) detects calcified nodules and microdissections. This imaging-guided approach allows selection of lesions with low likelihood of successful balloon predilation, thereby reducing the requirement for rRA. Second, an pRA first strategy should be prioritized over rRA to minimize periprocedural complications. Second, while pRA remains the preferred approach for severely calcified lesions, rRA demonstrates comparable long-term clinical outcomes and can be safely utilized as a bailout strategy when required by procedural complexity.

### Study limitations

This study is a single-centre retrospective study. The sample size is limited, which may bias the results. Multicentre randomized prospective controlled studies with large sample sizes are still needed for further verification of present results.

## Conclusion

Rescue RA is related with higher procedural complication rates, procedure time, and contrast agent dose compared with pRA, but has similar low MACCE rate as pRA at one year after procedure. Thus, our results indicate that rescue RA remains as a feasible clinical PCI strategy in indicated patients without safety concern in terms of long-term outcome.

## Data Availability

The original contributions presented in the study are included in the article/Supplementary Material, further inquiries can be directed to the corresponding author.
